# Cultivation of an Adaptive Domestic Network for Surveillance and Evaluation of Emerging Infections

**DOI:** 10.3201/eid2109.150619

**Published:** 2015-09

**Authors:** Robert W. Pinner, Ruth Lynfield, James L. Hadler, William Schaffner, Monica M. Farley, Mark E. Frank, Anne Schuchat

**Affiliations:** Centers for Disease Control and Prevention, Atlanta, Georgia, USA (R.W. Pinner, M.E. Frank, A. Schuchat);; Minnesota Department of Health, St. Paul, Minnesota, USA (R. Lynfield);; Yale University School of Public Health, New Haven, Connecticut, USA; (J.L. Hadler);; Vanderbilt University School of Medicine, Nashville, Tennessee, USA (W. Shaffner);; Emory University School of Medicine, Atlanta (M.M. Farley)

**Keywords:** public health, infectious diseases, emerging infections, epidemics, outbreaks, surveillance, Emerging Infections Program, EIP

## Abstract

Accomplishments of this program have provided numerous dividends and might benefit areas outside infectious diseases.

“The best time to plant a tree was 20 years ago; the second best time is now.”—Chinese Proverb

Through the metaphor of an adaptive, organic entity—a tree with roots, a trunk, large limbs and smaller branches, fruits, and seeds (Figure 1)—this article describes the Emerging Infections Program (EIP), reflects on this network’s accomplishments over the past 20 years, and considers opportunities and challenges for the future. Other articles in this 2015 20th anniversary issue of Emerging Infectious Diseases focusing on the EIP expand on many of the ideas introduced here, providing additional discussion, details, and references.

**Figure 1:**
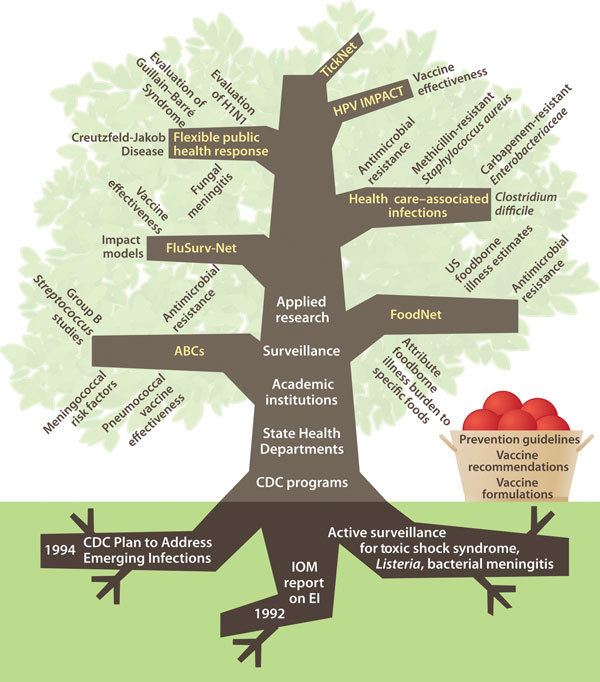
Structure and development of the Emerging Infections Program, United States. ABCs, Active Bacterial Core Surveillance; CDC, Centers for Disease Control and Prevention; IOM, Institute of Medicine; EI, emerging infections; HPV, human papillomavirus.

## Roots

The concepts of emerging infectious diseases are now familiar to the scientific community and the public. However, it took a 1992 Institute of Medicine report to emphasize the dynamic and modern factors that cause infectious diseases to emerge and re-emerge and to put to rest the idea of infectious diseases as a solved problem, a worry for earlier times (*1*). The Centers for Disease Control and Prevention (CDC) Plan to Address Emerging Infections, released in April 1994, provided recommendations for action by CDC and other public health agencies (*2*). The CDC Plan highlighted the foundational role of surveillance and included in the recommendations creation of a network comprising state public health agencies, academic institutions, and CDC for special surveillance and applied public health research. The EIP sprang from these recommendations.

Even before that time, active, population-based surveillance projects dating to the 1970s had provided a general model for the EIP. Active surveillance and related research conducted through collaborations between CDC and health departments generated information on the burden of and risk factors for toxic shock syndrome, listeriosis, *Haemophilus influenzae* type b (Hib) and group B *Streptococcus* (GBS) infections, and meningococcal disease (*3**–**6*). An earlier population-based active surveillance effort on bacterial meningitis conducted in Bernalillo County, New Mexico, provided a similar model (*7*). The approach—population-based, active, laboratory-based surveillance, sometimes coupled with collection of disease-causing isolates and always including key epidemiologic information—was incorporated into today’s EIP activities.

Whereas earlier activities focused on a single disease or a small number of diseases and activities and operated through contracts between CDC and health departments, from the beginning the EIP dealt with multiple public health issues concurrently; engaged experts in state public health agencies, academic institutions, and a variety of CDC programs; and operated as a consortium in which stakeholders have mutual responsibilities for setting priorities, planning and executing activities, and synthesizing and communicating results (*8**,**9*).

## Trunk

Understanding the urgency, challenge, and complexity of its mission and the need for a flexible model to support it, the EIP built a network of collaborator sites, each contributing to shared governance, and established a strategic approach to guide projects. These elements serve as the trunk, or supportive infrastructure, for EIP efforts.

**Figure 2:**
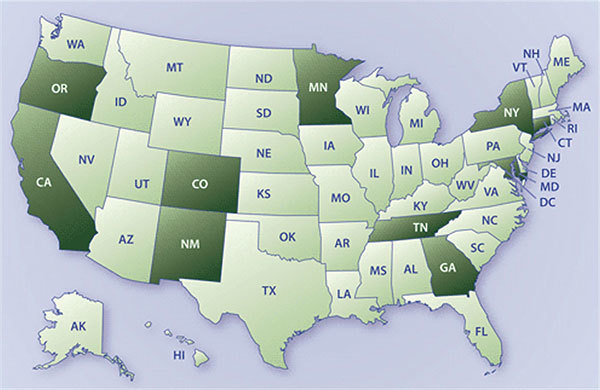
The Emerging Infections Program (EIP) and its key partnerships, United States. Dark shading indicates locations of EIP sites (year established are indicated in parentheses). Minnesota: Department of Health, St. Paul, and Association of Professionals in Health Control, St. Paul (1995); Oregon: Oregon Public Health Division, Portland, and Oregon Health Sciences University, Portland (1995); California: Department of Public Health, Sacramento, and University of California School of Public Health, Berkeley (1995); Colorado: Department of Public Health and Environment, Denver, and University of Colorado Health Sciences Center, Denver (2000); New Mexico: Department of Health, Santa Fe, and University of New Mexico Indian Health Service, Albuquerque (2002); New York: Department of Health, New York, and University of Rochester, Rochester (1997); Connecticut: Department of Public Health, Hartford, and Yale University School of Public Health, New Haven; Maryland: Department of Health and Mental Hygiene, Baltimore, University of Maryland, College Park, and Johns Hopkins University, Baltimore (1997);  Tennessee: Department of Health, Nashville, and Vanderbilt University, Nashville (1999); Georgia: Department of Public Health, Atlanta, Emory University School of Medicine, Atlanta, and Atlanta Veterans Administration Medical Center, Atlanta.

The number of sites increased—from 4 in 1994 to the current number of 10 by 2002—as EIP activities demonstrated success, the need for broader geographic and demographic representation was recognized, and funds became available (Figure 2). EIP sites involve state health department personnel and key collaborators in academic institutions; each site engages others to conduct activities, including clinical laboratories and infection control professionals throughout each EIP area. The 10 EIP sites, together with several CDC programs and a coordinating unit at CDC, form the EIP network. EIP support comes from core funding intended to maintain and support the network and invest in key activities. In addition, other sources support specific EIP activities. For example, funding from the US Department of Agriculture (USDA), the Food and Drug Administration (FDA), and the Food Safety Initiative of CDC have supported foodborne diseases work; the immunization program of CDC supports vaccine effectiveness evaluation and related surveillance of vaccine-preventable disease. Extramural funding for EIP cooperative agreements has ranged from $2.3 million for 4 sites in 1995 to an average annual total of $33.8 million for the current 10 sites during 2010–2014.

As early as the first EIP meeting in November 1994, principals at CDC and EIP sites (including representatives from state health departments and academic partners) formed an EIP Steering Group to provide guidance and strategy for EIP activities. By the time of the Steering Group meeting in November 1996, the group had adopted guiding principles and approved a framework for evaluating ideas for new projects, which has guided assessment of potential new areas of work and strategic directions (Table).

**Table:** Guiding principles for the Emerging Infections Program complied from notes of the meeting of the EIP Steering Group, November 13–14, 1996, United States*

Guiding principles
EIP network is a national resource for surveillance, prevention, and control of emerging infectious diseases. EIP activities go beyond the routine functions of health departments in ways that enable challenging new public health questions to be answered.
Core EIP activities target the most pressing issues in infectious disease and are selected with regard to what is appropriate, in particular, for the EIP network (including considerations such as the burden of disease, preventability, and providing resources not provided through categorical funding)
EIP maintains sufficient flexibility for emergency response and to address new problems as they arise.
Training is a key function of EIP (public health students, laboratory personnel, preventive medicine residencies, infectious disease fellows)
EIP network develops and evaluates public health practices and transfers what is learned to the public health community (e.g., computerized transfer of data, molecular epidemiology, accomplishing public health work successfully in a changing health care environment).
EIP network should give high priority to projects that lead directly to prevention of disease.

*EIP, Emerging Infections Program.

Responsibilities and authorities are distributed across the network’s membership. State public health agencies have legal authority for conducting surveillance; in this context, academic partners function as agents of the state health departments. CDC has responsibility for expending and managing federal funds invested in the EIP. Resources come from several funding streams, and each source requires accountability for ensuring that funds are spent well on appropriate activities. This distribution of responsibilities and authorities, coupled with the need for ensuring that the EIP can respond nimbly to emerging issues, has meant that governance works flexibly, not rigidly—through negotiations and consensus—for the network as a whole and also in each project area. If one considers the distribution of interests and authorities, this model has proven productive. In addition to internal governance, EIP work has benefited from external reviews that provided advice and guidance on strategic directions, and from representatives of professional organizations (e.g., Infectious Diseases Society of America, Society for Healthcare Epidemiology of America, Council of State and Territorial Epidemiologists, American Society for Microbiology) serving on several EIP steering committees.

EIP activities generally fall into the categories of surveillance, applied research, and enhanced and flexible public health practice. Active, population-based and laboratory-based surveillance, with collection of disease-causing isolates linked to epidemiologic information from case reports, forms the foundation of many EIP activities. This foundation accurately documents the burden of disease and key characteristics of disease-causing microbes and supports special applied research activities, such as evaluation of vaccine effectiveness and epidemiologic risk factor studies. On several occasions, the EIP has proved its flexibility and provided enhanced responses to precipitously emerging issues.

## Limbs and Branches

With an established network of sites, governance, and a strategy in place, the main limbs, or programs, of the EIP grew in 4 broad thematic areas: invasive bacterial diseases; foodborne diseases; health care–associated infections; and influenza. Each program contains a portfolio of established and newer projects. Leveraging EIP resources flexibly as needed to provide fast public health responses to emerging outbreaks is a fifth limb of the EIP tree. Other branches fill out the tree.

### Active Bacterial Core Surveillance

Active Bacterial Core Surveillance (ABCs) of the EIP determines the incidence and epidemiologic characteristics of invasive disease caused by bacterial pathogens, including *Streptococcus pneumoniae*, groups A and B *Streptococcus*, *H. influenzae*, *Neisseria meningitidis*, and *Bordetella pertussis* (*10**,**11*). ABCs activities comprise surveillance and studies to better understand diagnostics, risk factors for disease, and vaccine effectiveness.

### Foodborne Diseases Active Surveillance Network

The Foodborne Disease Active Surveillance Network (FoodNet), the principal foodborne disease component of the EIP, is a collaborative venture among the 10 EIP sites, the USDA and the FDA. This network monitors foodborne disease caused by bacterial and parasitic pathogens (*Campylobacter*, *Cryptosporidium*, *Cyclospora*, *Listeria*, and *Salmonella* spp.; Shiga toxin–producing *Escherichia coli* O157 and non-O157 *E. coli*; and *Shigella*, *Vibrio*, and *Yersinia* spp.) (*12*).

### Influenza Hospitalization Surveillance Network

Through the Influenza Hospitalization Surveillance Network, the EIP, along with additional states, conducts surveillance for laboratory-confirmed influenza-related hospitalizations in children and adults (*13*). The influenza program at CDC uses this surveillance information from EIPs, together with surveillance for other aspects of influenza to develop a full annual picture of influenza and the effect of vaccination efforts in the United States.

### Healthcare-Associated Infections Community Interface

The Healthcare-Associated Infections Community Interface (HAIC) investigates major and time-sensitive questions about emerging health care–associated infection (HAI) threats and antimicrobial drug resistance in the United States. The in-depth approach of the EIP to surveillance that monitors HAI diseases in health care institutions and the community and related research activities complements the broader approach used by the National Healthcare Safety Network (*14*).

### Other Branches

Other EIP branches include earlier projects on unexplained deaths, encephalitis, hepatitis, and current TickNET and human papillomavirus (HPV) IMPACT projects (*15*). Surveillance to identify causes for unexplained deaths with characteristics of infectious diseases was conducted during the early years of the EIP (*16*). Subsequently, EIP investigators in some sites focused on the clinical challenges in diagnosing encephalitis and resultant difficulties in epidemiologic characterization, and undertook a several-year project on encephalitis. Beginning by comparing and validating several diagnostics tests, this project estimated the burden and honed characterizations of encephalitis syndromes in relation to causative agents (*17*). TickNET is a network of 5 EIP sites created in 2007 to foster collaboration on surveillance, research, education, and prevention for tickborne diseases. HPV IMPACT conducts a postlicensure evaluation of HPV vaccine in 5 EIP sites (*18*).

### Flexible Responses to Emerging Issues and Outbreaks

Flexibility to respond is a foundational principle for the EIP. There are several examples of the EIP’s timely engagement in urgent situations.

#### Creutzfeldt-Jakob Disease, 1996

In 1996, an expert committee to the government of the United Kingdom recognized cases in humans of a new variant Creutzfeldt-Jakob disease (CJD) and concluded that the agent responsible for bovine spongiform encephalopathy might have spread to humans. The EIP then rapidly developed active CJD surveillance in 5 sites. This surveillance, coupled with other reviews of national CJD mortality rates, provided some assurance that the new variant CJD had not spread to the United States and helped substantiate effectiveness of death certificate reviews in identifying CJD deaths in the United States (*19*).

#### Hib Vaccine Shortage, 2008

When an Hib vaccine shortage occurred in the United States during 2008, the EIP contributed to evaluating the potential effect of deferred doses through active surveillance in the ABCs. In addition, EIP sites in Georgia and Minnesota evaluated nasopharyngeal carriage of Hib (*20**,**21*).

#### Influenza A(H1N1)pdm09, 2009

The EIP made contributions during the influenza pandemic in 2009, not only through surveillance of hospitalizations caused by influenza but also by conducting a key evaluation of vaccine safety during the immunization campaign that year. Because of the prior association between Guillain-Barre syndrome and the 1976 vaccine against H1N1 subtype influenza virus, the EIP was engaged to conduct enhanced surveillance to estimate the magnitude of any increased risk for Guillain-Barre syndrome after administration of vaccine against influenza A(H1N1)pdm09 virus. The EIP findings, that the excess risk was comparable with that associated with prior seasonal influenza vaccines and smaller than that observed in 1976, provided evidence for sustaining the vaccination campaign (*22*).

#### Fungal Meningitis Epidemic, 2012

Beginning in 2012, Tennessee EIP staff first detected and then provided leadership in a multistate investigation of fungal meningitis. This outbreak was caused by use of contaminated medication and resulted in 751 cases and 64 deaths across 20 states (*23**,**24*).

## Fruits

The EIP has borne fruit in several areas. These areas include postlicensure evaluation of vaccines, foodborne diseases, antimicrobial resistance, and health care–associated infections. The EIP has communicated its findings in nearly 1,000 publications.

### Vaccine Development and Policy

The EIP has provided critical elements of the evidence base to support US immunization policy, including addressing the burden of disease, defining population groups at higher risk, evaluating cost-effectiveness of various vaccine recommendations, and determining duration of protection after widespread use. Initial recommendations for 7-valent pneumococcal conjugate vaccine (PCV7), 13-valent pneumococcal conjugate vaccine, and meningococcal conjugate A/C/Y/W-135 vaccines were supported by ABCs data, and the HPV IMPACT project provided outcome data that helped evaluate early effects of HPV vaccine implementation (*10**,**25**,**26*). The EIP’s laboratory-based surveillance and characterization of circulating strains contributed to development and recent recommendation for use of meningococcal B vaccines and group A streptococcal vaccines under development (*27**,**28*).

### Formulating, Implementing, and Evolving an Effective Public Health Prevention Strategy against Perinatal GBS Disease

A series of surveillance and prevention studies from ABCs showed the preventable burden of early-onset (GBS) infections, evaluated the relative effectiveness of initial screening vs. risk-based prevention strategies, provided assessments of prevention guidelines uptake and effect, and identified missed opportunities for additional prevention. A retrospective cohort study (*10*) conducted by using ABCs infrastructure showed that prenatal screening was 50% more effective than the risk-based strategy of directing intrapartum antimicrobial prophylaxis. These data directly resulted in revised GBS prevention guidelines by providing compelling evidence for the recommendation to implement universal prenatal GBS screening. Application of GBS prevention strategies in the era of the EIP has contributed to prevention of >85,000 early onset GBS cases (*10*).

### Guiding and Monitoring Food Safety Efforts

EIP FoodNet has provided standard surveillance data used by federal agencies—including the FDA, the USDA, and CDC—to assess national trends and progress in reducing foodborne diseases caused by bacterial and parasitic pathogens (*12*), especially in the context of implementing the Food Safety Initiative in 1997 and, more recently, the Food Safety Modernization Act in 2011. Studies conducted at FoodNet sites have also provided many data that contributed to estimates of the burden of foodborne pathogens in the United States in 1999 and in 2010 (*12*). In 1999, studies of antimicrobial drug resistance in *Campylobacter* spp. provided data connecting fluoroquinolone use in animals with emerging fluoroquinolone resistance in human cases of campylobacteriosis (*29*). The FoodNet Population Survey has produced a periodic atlas of specific food consumption prevalence in EIP sites (*12*). The atlas has not only provided baseline data to guide and monitor food safety educational efforts but has become a standard source of data for identifying suspect food in outbreaks caused by widely distributed foods (*9**,**12*).

### Investigating and Responding to Antimicrobial Resistance and Health Care–Associated Infections

Over the past 2 decades, the EIP has strengthened the evidence base regarding several antimicrobial drug–resistant pathogens. EIP projects contributed data to the CDC report on Antibiotic Resistance Threats in the United States, 2013, a widely publicized report that outlined the extent of the public health threat of antimicrobial drug resistance (*30*). This report helped prompt development of a National Strategy to Combat Antimicrobial Resistance in Bacteria, issued in March 2015 (*14*).

The EIP has studied antimicrobial drug resistance in invasive pneumococcal disease, methicillin-resistant *Staphylococcus aureus* (MRSA), *Clostridium difficile*, carbapenem-resistant *Enterobacteriaceae*, infections with *Candida* species, and patterns of antimicrobial drug use. The program documented a decrease in drug-resistant invasive pneumococcal isolates after widespread use of PCV7; emergence of resistant serotype 19A, which was not included in PCV7; and another decrease in drug-resistant pneumococcal disease after use of 13-valent pneumococcal conjugate vaccine, which included 19A (*30**,**31*). Analysis of outpatient drug prescriptions and ABCs data found that high use of antimicrobial drugs was correlated with the proportion of nonsusceptible invasive pneumococcal disease, which suggested that local prescribing practices contribute to local drug resistance patterns (*32*). The EIP was instrumental in describing the emergence of community-associated MRSA (*30*), the burden of invasive MRSA (*10*), and a decrease in rates of health care–associated MRSA (*33*). The network determined the burden of infections with *C. difficile* (*34*) and established surveillance for carbapenem-resistant *Enterobacteriaceae* (*35*). Finally, because antimicrobial drug resistance is driven by use of these drugs, the EIP has conducted prevalence surveys to determine the frequency of infections and use of these drugs in hospitals (*14*).

## Seeds

The EIP has planted seeds in the United States and abroad. EIP training, consultation, and collaboration activities have made substantial contributions to public health efforts.

### Training in the United States

The EIP has engaged many health care professionals in training, among them numerous master’s-level and doctoral-level students. These students have worked on EIP projects that have fulfilled the thesis or practicum requirement for their degree, and many have resulted in publications in peer-reviewed journals and public presentations. In addition, EIP site personnel provide scientific presentations and updates on emerging infectious diseases to local health and public health partners, and several EIP sites hold annual conferences and symposia in their regions (*36*).

### EIP-Like Activities Abroad

Surveillance methods, study protocols, and results of EIP work have had effects around the world. An integrated infectious disease and specimen characterization surveillance system in South Africa, modeled after ABCs, has provided valuable information on invasive bacterial, diarrheal, and fungal infections and the effect of pneumococcal and Hib vaccines, and on decreasing opportunistic infections in conjunction with antiretroviral treatment among HIV-infected populations. Data from the ABCs PCV7 vaccine effectiveness study conducted when a vaccine shortage resulted in substantial numbers of children receiving <4 doses of vaccine provided information on partial schedules that supported licensure of 3-dose schedules in the United Kingdom and other countries. Economic analysis that incorporated indirect and direct effects of PCV, derived from EIP data, provided pivotal information for vaccine introduction decisions in countries where initial assessments, before recognition by ABCs investigators that there were indirect benefits, had led policy makers to conclude that the vaccine was too costly to be used routinely. The EIP model spawned International EIPs in Thailand and Kenya (*37*) and was adapted later to regional Global Disease Detection Centers established by CDC and ministries of health in other countries.

## Changes in the Climate for EIP

Whereas weather changes often—hourly, daily, and seasonally—climate changes occur more slowly but may have profound effects. From its origins, the EIP has been in the habit of responding flexibly to the severe weather of outbreaks and emerging diseases. Now, however, the broader scientific, technological, and cultural climate in which public health agencies operate and in which emerging infections are addressed is changing substantially, requiring the EIP to adapt.

### Culture-Independent Diagnostic Tests and Advanced Molecular Detection

EIP active surveillance for bacterial diseases has depended on isolation of the disease-causing organism. Case finding started in clinical laboratories, and case definitions have included isolation of an organism as part of the case definition (e.g., invasive pneumococcal disease—isolation of *S. pneumoniae* from a normally sterile body site). Clinical diagnoses are increasingly being made through culture-independent diagnostic test (CIDTs), particularly nucleic acid–based tests. Although CIDTs might represent advances in modern medical practice, they can also confound EIP surveillance. Culture-independent diagnostic tests vary in their performance characteristics, and also their market share across EIP sites, which can influence incidence measurements, potentially causing discontinuity of data or requiring modeling to estimate incidence in a way that has not been previously needed. Moreover, the EIP has relied on isolates for antimicrobial drug–susceptibility testing and molecular epidemiology, which cannot be conducted—or conducted in the same way—if there are no longer clinical isolates. EIP surveillance methods, analytic methods, and case definitions will need to adapt, as will laboratory methods applied for drug susceptibility and molecular typing in EIP projects.

Even as CIDTs might challenge the continuity and quality of surveillance data, advances in laboratory technology also present new opportunities. For example, the EIP is engaged in the new advanced molecular detection (AMD) initiative at CDC to explore and advance application of modern molecular technologies to the practice of public health. With its huge asset of collections of population-based and epidemiologically well-characterized strains, the EIP is well positioned to apply AMD methods, such as whole-genome sequencing and metagenomics. As the EIP applies these powerful new tools to characterize strains and understand pathogenesis, they will enhance the quality of the network’s science and contribute to the transformation of public health practice that the AMD initiative provides (*38*).

### Information Technology and Electronic Health Records

Systematic review of paper medical records by EIP surveillance officers has been central in developing high-quality information for EIP surveillance and special studies. As electronic health records evolve, this historical approach is disappearing and new efforts by EIP staff are required to gain appropriate and ready access to electronic records and new skills are needed to use them effectively. However, the potential for more efficient, powerful, and innovative use of modern health information technology can outweigh the problems caused by the transition from paper to electronic health records. Instead of transcribing data from charts into EIP surveillance and study forms, well-structured outputs from electronic records can save substantial staff time and resources. Also, use of structured or even ad hoc queries could make EIP surveillance and research projects more flexible and powerful. For example, EIP HAI surveillance uses queries of laboratory-automated culture and susceptibility systems to identify patterns that fit the case definition of multidrug-resistant *Enterobacteriaceae*. Moreover, modern geographic information systems technology offers tremendous possibilities for complementing disease surveillance with monitoring distribution of disease vectors. Recently, the EIP has identified a standard approach for geocoding cases. Adoption of this approach across EIP projects will enable researchers to connect information about cases from different EIP projects (e.g., influenza and pneumococcal pneumonia), which, when linked with other geospatial data, such as socioeconomic or climate or land use data, might help clarify underlying determinants of health and health disparities and the extent to which these pathways are similar across different diseases.

### Health Reform and Public Health Practice

Health reform in the United States is affecting the way persons are obtaining health care and is also influencing the range of preventive services available, how they are delivered, and how they are funded. As the relationship between clinical care and public health evolves, there might be a role for the EIP in filling scientific gaps at the population level. The EIP could participate in assessment of the effect of health care reform on health department infectious disease control practice (e.g., evaluation of the role of health departments in direct delivery of clinical services for infectious diseases, such as immunization for tuberculosis and sexually transmitted diseases).

## Conclusions and New Directions

The EIP model—close collaboration among state and federal public health agencies along with academic institutions and generation of reliable surveillance information coupled with special studies to address key policy and prevention issues that generally use a population-based approach—has provided numerous dividends for public health work in infectious diseases. The EIP tree is flourishing.

Public health issues other than infectious diseases might also benefit from the EIP model. For example, opioid overdose in the United States, with its recent epidemic-like emergence, might be one such issue. During the coming year, the EIP will explore this idea through projects at 2 sites aimed at strengthening the scientific base for prevention of opioid overdose.

A central premise of the Institute of Medicine report on emerging infections was that the emergence and reemergence of infectious diseases are a consequence of dynamic processes and factors: societal events; health care; food production; human behavior; environmental changes; public health infrastructure; and microbial adaptation (*1**,**2*). Taking these factors into account, the EIP developed into a productive, flexible, and adaptive public health and scientific network. Although current circumstances differ substantially from when the network was founded, in challenges to the public’s health and in tools to address them, this vision of an adaptive EIP remains apt. The aim of practicing consequential epidemiology has motivated persons who have engaged in the EIP; we hope this tenet will also guide another generation of public health professionals who will cultivate the EIP over the next 20 years (*39*).
